# A Time‐Programmed Bilayer Wound Dressing for Dynamic Microenvironment Modulation and Full‐Thickness Regeneration in Diabetic Wounds

**DOI:** 10.1002/advs.202512425

**Published:** 2026-01-08

**Authors:** Lei Yi, Wanqian Li, Ying Duanmu, Zihan Zhang, Shixuan Chen, Shichu Xiao, Lei Du, Miaomiao Wei

**Affiliations:** ^1^ Department of Burn Ruijin Hospital Shanghai Jiao Tong University School of Medicine Shanghai China; ^2^ Zhejiang Engineering Research Center for Tissue Repair Materials Wenzhou Institute University of Chinese Academy of Sciences Wenzhou Zhejiang China; ^3^ Department of Burn and Plastic Surgery Department of Wound Repair Surgery Affiliated Hospital of Nantong University Nantong Jiangsu China; ^4^ Department of Burn Surgery The First Affiliated Hospital of Naval Medical University Shanghai China

**Keywords:** growth factors, polyphenol, remodeling, wound healing, wound microenvironment

## Abstract

Chronic diabetic wounds suffer from dysfunctional repair programs due to accumulated advanced glycation end products (AGEs) and persistent inflammation in hyperglycemic microenvironments, posing significant clinical challenges. Current dressings predominantly focus on monofunctional interventions, failing to resolve the “inflammation‐repair imbalance”. In this study, we propose a chronotherapeutic bilayer system designed for stage specific regulation. The lower GelMA cryogel loaded with polyphenols acts as a “cleaner”, removing AGEs via hydrogen bond capture and pore entrapment and activating the PPARγ pathway to remodel the anti‐inflammatory microenvironment; The upper polycaprolactone nanofibers directionally deliver PDGF‐BB, enabling sustained release to activate fibroblast migration and angiogenesis. Additionally, proteomics analysis further revealed that polyphenols downregulate multiple inflammatory factors via the PPARγ/NF‐κB axis, providing a clean basal microenvironment for diabetes. In a diabetic wound model, the system demonstrated a three‐step healing, marked downregulation of inflammatory markers within 72 h, accelerated re‐epithelialization in 7 days, and functional hair follicle regeneration in 21 days. This study highlights the potential of “Clean‐Regeneration Dual Program” bilayer assembled scaffolds in modulating the wound microenvironment and promoting tissue regeneration.

## Graphical abstract

The Polyphenol+PDGF‐BB scaffold fabrication (Panel A) and its dual wound healing effects (Panel B: anti‐inflammation and accelerated tissue remodeling).

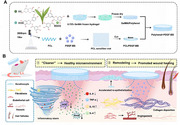

## 1 Introduction

Diabetes affected an estimated 828 million adults globally in 2022, with prevalence continuously rising [1]. Alarmingly, 445 million adults aged 30 years or older with diabetes remain untreated with oral hypoglycemics or insulin, and poor glycemic control can lead to a range of complications [2]. Among them, diabetic foot ulcers (DFUs) represent a severe complication of diabetes mellitus, with impaired healing rooted in abnormal epithelial regeneration during wound healing [3]. EURODIALE studies have shown that epithelial regeneration time ranges from 147 to 237 days [4]. In addition, high rates of ulcer recurrence (ranging from 7.7% to 44%) and increased lower limb amputations underscore the urgent need for improved DFU management strategies [5, 6].

Current common clinical approaches include dressing changes, debridement, and negative pressure suction [7]. State of inflammatory microenvironment at the wound base directly determines wound repair activity and healing rate [8]. Therefore, understanding the matrix microenvironment is critical for diabetic wound repair. Elevated glucose levels drive excessive protein glycosylation, forming AGEs that induce oxidative stress, sustained pro‐inflammatory cytokine expression, and extracellular matrix (ECM) dysfunction [9, 10]. Therefore, targeting AGEs accumulation or AGEs/ RAGE binding could fundamentally mitigate inflammation [11, 12, 13]. However, therapeutic modulation of the AGEs/ RAGE axis remains underexplored in chronic diabetic wounds.

Polyphenolic compounds have natural anti‐inflammatory and antioxidant properties [14], and studies have shown that they can modulate the inflammatory response through multiple pathways by inhibiting AGEs production, promoting AGEs inactivation, or interfering with AGEs/ RAGE binding [15, 16, 17]. However, most polyphenols suffer from high effective concentrations and cytotoxicity [18]. Furthermore, traditional Chinese medicine extracts typically possess complex compositions. For instance, green tea contains dozens of phenols, amino acids, alkaloids, and other components, with the biological functions of minor components yet to be fully elucidated [19]. Studies have shown that EGCG, ECG, and EC are the bioactive components in green tea extracts with the highest content [20, 21]. To enhance its therapeutic potential, we engineered a low‐toxicity, high‐efficiency cryogel by encapsulating this polyphenol within GelMA. GelMA possesses unique physicochemical properties that enable the conversion from a lamellar structure to aligned cryogel fiber scaffolds (ACFS) via directional freeze casting; moreover, its fiber structure can be precisely regulated to match the cell recruitment and tissue regeneration requirements of diabetic wounds. This not only reduces material dosage to minimize the wound foreign body reaction but also forms a looser fiber network through a low concentration system, reserving sufficient space for cell migration [22, 23]. One of the core bottlenecks in diabetic wound repair is the low efficiency of cell recruitment and disordered cell migration. GelMA addresses this issue directly by allowing the customization of fiber size, density, and alignment through regulating process parameters (e.g., freezing temperature, solution concentration). Additionally, GelMA enables stable hydrogen bond formation with polyphenols, allowing ACFS to achieve directional drug release while retaining its aligned structure [24].

Notably, growth factor‐mediated proliferation and angiogenesis are essential for DFU repair [25, 26]. PDGF‐BB is approved by the U.S. Food and Drug Administration for DFUs [27], plays a key regulatory role in both the inflammatory and proliferative phases, and has been widely used in a variety of acute and chronic trauma model studies [28]. Our team prior work demonstrated that PDGF‐BB loaded nanofiber microsphere scaffolds enhance fibroblast and endothelial cell recruitment and had excellent performance in chronic infected wound repair [29]. PCL nanofibrous membranes constructed via electrospinning replicate the ECM architecture, providing a biomimetic microenvironment for fibroblast and endothelial cell adhesion and proliferation [30]. Here, we constructed PDGF‐BB‐loaded PCL electrospun nanofibers. PDGF‐BB encapsulated in the organic phase was dispersed in the fiber matrix as nanoscale droplets, avoiding aggregation and realizing uniform loading at the molecular level.

**FIGURE 1:**
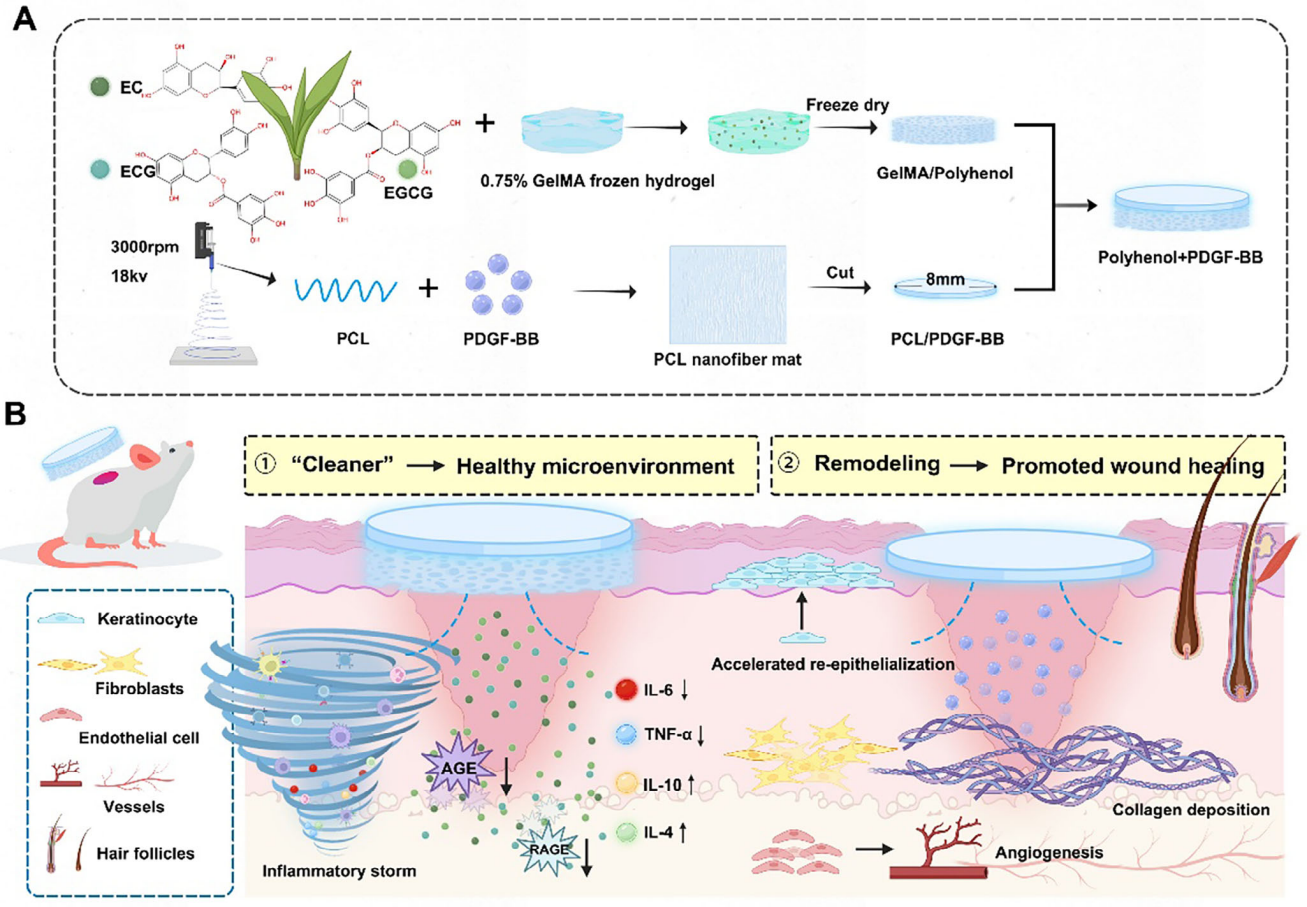
Schematic representation of wound healing mediated by Polyhenol+PDGF‐BB. (A) Preparation of GelMA/Polyphenol, PCL/PDGF‐BB, and Polyphenol+PDGF‐BB. (B) A two‐step mechanism of Polyphenol+PDGF‐BB to promote diabetic wound healing.

Taken together, we hypothesize that the bilayer system (Polyphenol+PDGF‐BB) achieves precision regulation throughout the wound healing procedure: (i) GelMA/Polyphenol targets AGEs/RAGE axis at the inflammatory source, reprogramming the chronic diabetic microenvironment; (ii) Upon inflammation resolution, the PDGF‐BB‐loaded PCL nanofibrous membrane rapidly recruits fibroblasts, endothelial cells, and keratinocytes to drive remodeling (Figure 1). In the presented study, we combined sequencing analyses to evaluate the ability of polyhenol as a “cleaner” and the potential of double‐layer dressings in diabetic wounds to programmatically restore a healthy wound microenvironment and full regeneration of skin tissue.

## 2 Result and Discussion

### 2.1 Preparation and Characterization of GelMA/Polyhenol and PCL/PDGF‐BB

**FIGURE 2:**
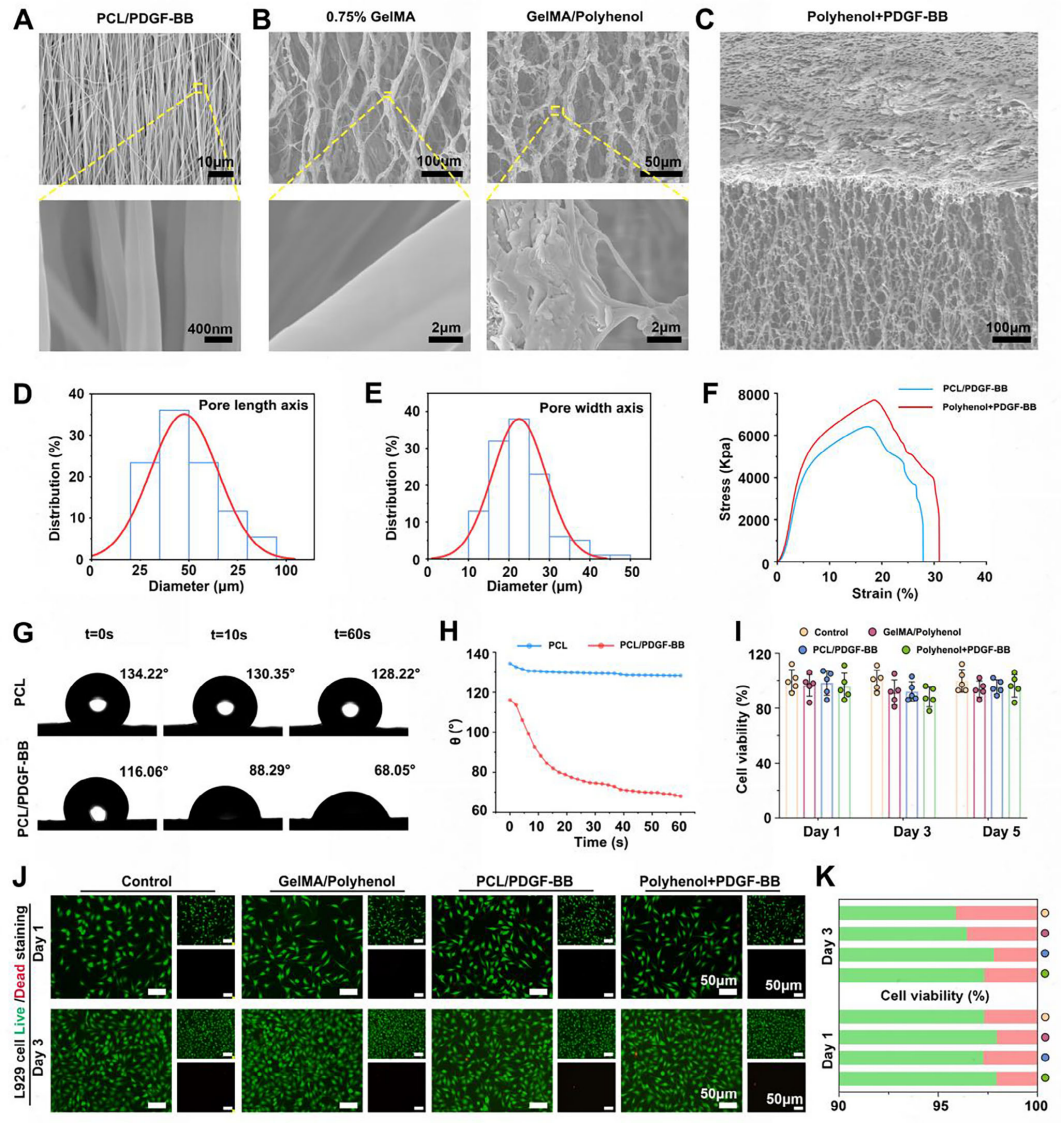
Synthesis, characterization, and biocompatibility of Polyphenol+PDGF‐BB. SEM images of (A) PCL/PDGF‐BB, (B) 0.75%GelMA, GelMA/Polyphenol, and (C) Polyphenol+PDGF‐BB. (D,E) Quantitative distribution analysis of the long and wide axes of GelMA/Polyphenol pore sizes. (F) Mechanical properties (stress–strain curve). (G) Contact angle experiments and (H) quantification of time‐dependent curves. (I) CCK8 results quantified on day 1, 3, and 5. (J,K) Live/ dead staining of L929 in Control, GelMA/Polyphenol, PCL/PDGF‐BB, and Polyphenol+PDGF‐BB groups on day 1 and day 3, followed by quantitative analysis. Scale bar: 50 µm. n = 5, error bars represent mean ± SD.

SEM revealed that the PCL/PDGF‐BB nanofiber fibers diameter was about 200 nm and showed a directional distribution (Figure 2A). The 0.75% GelMA layer, crosslinked via APS and TEMED initiated radical polymerization, developed a porous network after lyophilization. This structure can adsorb wound exudate while providing a microenvironment for fibroblasts and keratinocytes to adhere and proliferate, guiding neovascularization and tissue remodeling. SEM observed that the 0.75% GelMA cryohydrogel showed a porous network structure, in contrast to GelMA/Polyphenol, which exhibited a significant reduction in fiber pore size, and a significant amount of polyphenol loaded on the fibers could be seen in the magnified image (Figure 2B). Polyphenols were incorporated via physical adsorption and network entrapment, enabling sustained release upon fluid absorption at the wound site. This mechanism minimizes burst release, avoiding local overdose or unnecessary waste. Figure 2C shows the SEM microstructure of the Polyhenol/PDGF‐BB bilayer dressing. Quantitative analysis demonstrated that the incorporation of polyphenols reduced the pore size (long axis:47.59 ± 17.45 µm, width axis:18.79 ± 8.09 µm) (Figure 2D,E). Polyphenols exhibited a burst release phase from 0 to 24 h, with a cumulative release rate of 48.4% ± 3.6% at 24 h. Release plateaued after 72 h, meeting the demand for rapid early‐stage inflammation control. PDGF‐BB entered a sustained‐release phase after 1 day, reaching peak release between 3 and 7 days. The cumulative release rate was 53.6% ± 5.6% at 7 days and 85.4% ± 4.6% at 14 days, continuously promoting regeneration during the late wound healing stage (Supplementary Figure S1A,B). The tensile stress–strain curve indicates that the electrospun loaded polyphenol+PDGF‐BB composite exhibits a maximum tensile stress of 7338.93 ± 364.39 kPa and an elastic modulus of 1124.66 ± 72.87 kPa. while the PCL/PDGF‐BB composite exhibited a maximum tensile stress of 6712.3266 ± 315.86 kPa and an elastic modulus of 1003.59 ± 70.69 kPa (Figure 2F). The elastic modulus is comparable to that of human skin (5 kPa to 140 MPa), meeting the mechanical performance requirements for materials during daily human activities and ensuring the durability of the dressing during use. The contact angle of water droplets on PCL and PCL/PDGF‐BB surfaces changes over time. The contact angle on PCL/PDGF‐BB rapidly decreased from 116.06° to 68.05°, indicating that the addition of PDGF‐BB significantly enhances the material's hydrophilicity (Figure 2G,H).

### 2.2 In Vitro Biocompatibility Evaluation

For biomedical applications, it is crucial that materials not only perform their intended functions but also exhibit excellent biocompatibility [31]. To assess cytotoxicity in *vitro*, we employed the CCK‐8 assay and Live/ Dead staining to evaluate the cell compatibility of the Polyhenol/PDGF‐BB. L929 fibroblasts were co‐cultured with GelMA/polyphenol, PCL/PDGF‐BB, and Polyphenol/PDGF‐BB. As shown in Figure 2I, CCK‐8 results indicated high cell viability in all groups, maintaining an unaffected proliferative capacity during 1, 3, and 5 days of co‐culture. Live/Dead staining results (Figure 2J,K) further corroborated these findings, demonstrating that the majority of cells remained viable and exhibited healthy morphology after 1 and 3 days of incubation.

### 2.3 In Vivo Anti‐Inflammatory and Pro‐Regenerative Effects of Polyphenol

**FIGURE 3:**
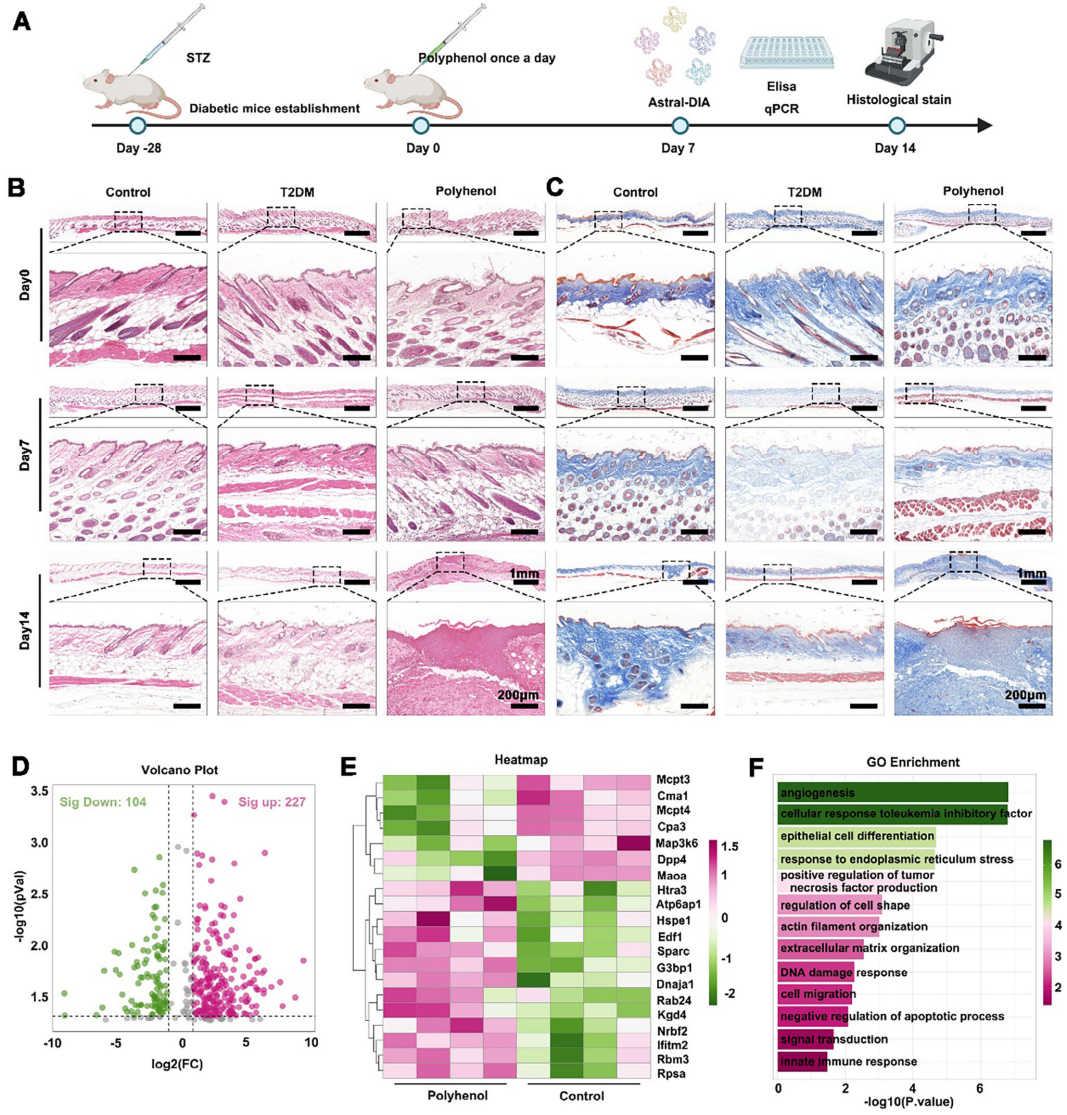
Anti‐inflammatory and repair ability of Polyhenol in type 2 diabetes mellitus and DIA quantitative proteomic analysis. (A) Timeline of establishment, treatment, and evaluation of type 2 diabetic mice. (B) Representative images of H&E staining at on days 0, 7, and 14. (C) Representative images of Masson's trichrome staining on days 0, 7, and 14. (D,E) Volcano and heat maps showed differentially expressed genes in the polyhenol group compared to the Control group. (F) GO functional enrichment analysis (Biological Process).

Chronic inflammation in diabetic wounds stems from AGEs and cytokine deposition [32]. These factors continuously stimulate overactivation of macrophages and neutrophils, triggering a persistent “inflammatory storm” [33]. Previous studies have shown that polyhenol inhibits excessive inflammation in chronic wounds through multi targets and multiple pathways, and plays a “cleaner” role in the process of wound repair [34]. Therefore, after 14 days subcutaneous polyphenol injection (Figure 3A), H&E and Masson staining revealed a significant reduction in cutaneous inflammation (Figure 3B,C). On day 14, skin tissue from diabetic mice exhibited interstitial edema, disorganized and sparsely distributed collagen, and decreased eosinophilic staining of tissues, suggesting deposition of protein glycosylation compared with healthy mouse tissues. However, polyphenol‐treated diabetic skin displayed reduced AGEs deposition, orderly collagen fiber alignment. To further explore these observations, skin tissue from the injection site was subjected to DIA‐based quantitative proteomic analysis (n = 4 per group). A total of 6934 proteins were identified, and the volcano plot showed 331 significantly differentially expressed proteins, including 227 up‐regulated proteins and 104 down‐regulated proteins (Figure 3D). Heat map clustering confirmed the reversal of diabetes‐related protein expression patterns in the polyphenol group (Figure 3E). Most proteins downregulated after polyphenol treatment are associated with inflammatory activation and metabolic disorders. Mcpt3, Cma1, Mcpt4, and Cpa3 are all molecules related to mast cell degranulation, and excessive mast cell activation is a major contributor to chronic inflammation in diabetic wounds. NF‐κB serves as a key regulatory pathway for mast cell inflammatory activation. Polyphenols downregulate these molecules, thereby reducing mast cell‐mediated inflammatory cytokine release and alleviating wound inflammation. Downregulating Map3k6 inhibits the activation of the NF‐κB pathway and reduces the production of pro‐inflammatory cytokines. Dpp4 involved in glucose metabolism and inflammation regulation; its overexpression exacerbates metabolic disorders and inflammation in diabetes. The downregulation of these proteins directly points to the role of polyphenols inhibiting inflammation and improving metabolic disorders in diabetic wounds. Htra3, Atp6ap1, and Hspe1 regulate cell proliferation and survival, their upregulation enhances the proliferative activity of wound cells.

GO enrichment analysis results for “positive regulation of tumor necrosis factor production”, “innate immune response”, and “negative regulation of apoptotic process” indicate that polyphenols can inhibit excessive activation of innate immunity, reduce abnormal cell apoptosis, alleviate chronic inflammation in diabetic wounds, and create a non‐inflammatory microenvironment for repair. Furthermore, through the enrichment of “angiogenesis”, “extracellular matrix organization”, “cell migration”, and “negative regulation of apoptotic process”, polyphenols activate angiogenesis related pathways, promote the formation of new blood vessels, improve the repair impairment caused by ischemia and hypoxia in diabetic wounds, and facilitate tissue regeneration (Figure 3F; Supplementary Figure S2A,B).

In addition, GSEA plots for PPAR and NF‐κB revealed significant positive enrichment of PPAR‐related gene sets and significant negative enrichment of NF‐κB‐related gene sets (Supplementary Figure S2C,D). This directly confirms the targeted regulatory trend of polyphenols on the pathways. Moreover, we further verified by detecting *Pparγ* (the gene encoding PPARγ) and *Nfkb1* (the gene encoding a key subunit of NF‐κB) in tissues. The results showed that in the polyphenol group, the mRNA expression of *Pparγ* was upregulated by 1.82 ± 0.30‐fold (*p* < 0.01), and the mRNA expression of *Nfkb1* was downregulated by 2.04 ± 0.32‐fold (*p* < 0.01) (Supplementary Figure S2E).

### 2.4 Polyphenols Scavenge AGEs and Inflammatory Factors In Vivo

**FIGURE 4:**
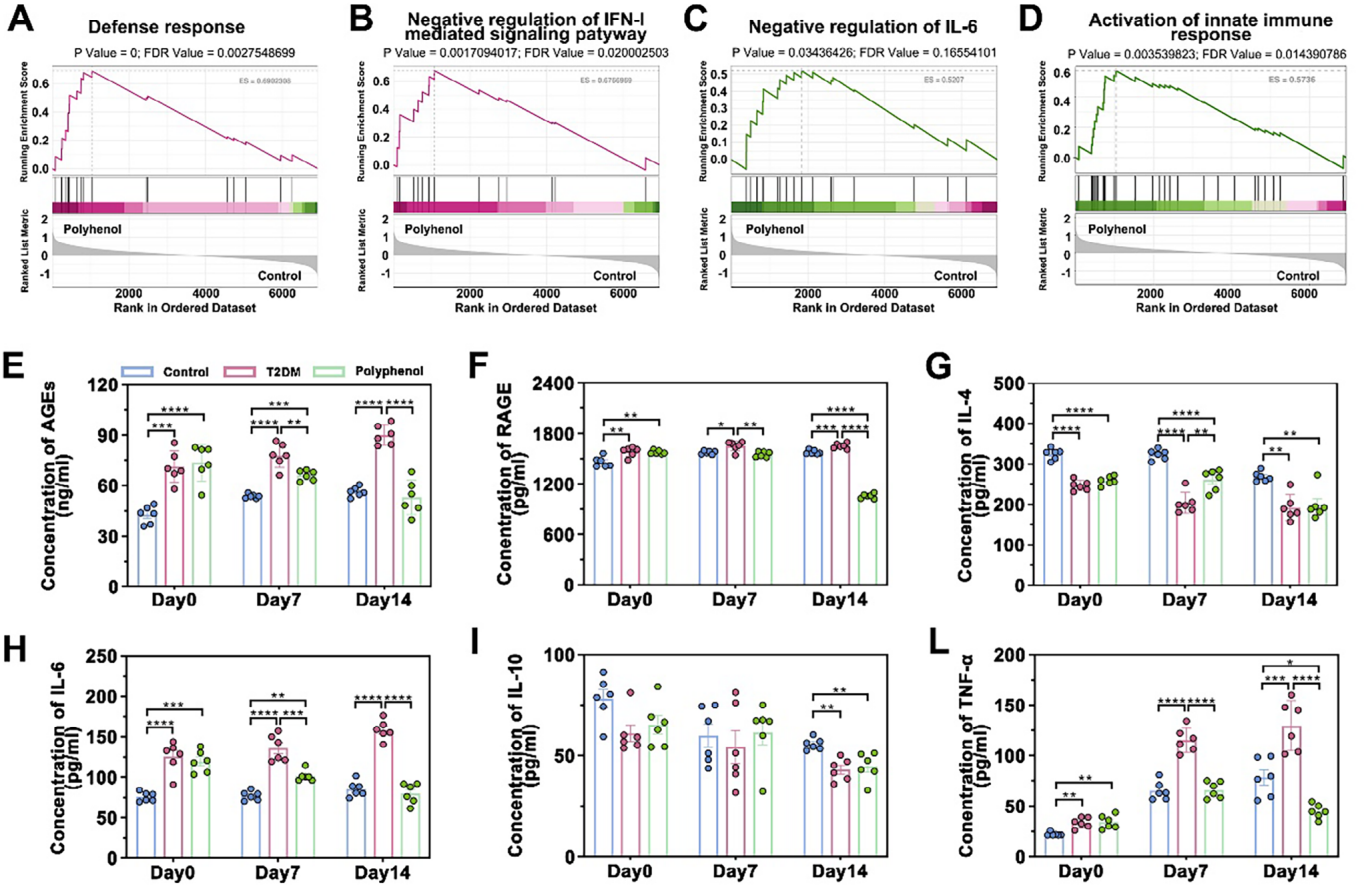
Polyphenols scavenge AGEs and inflammatory factors. Gene set enrichment analysis (GSEA) of (A) defense response, (B) negative regulation of IFN‐I mediated signaling pathway, (C) negative regulation of IL‐6, and (D) activation of innate immune response. Differences in (E) AGEs, (F) RAGE, (G) IL‐4, (H) IL‐6, (I) IL‐10, and (L) TNF‐α levels between groups Control, T2DM, and Polyphenol were detected by Elisa. ^*^ *p* < 0.05, ^**^ *p* < 0.01, ^***^ *p* < 0.001, ^****^ *p* < 0.0001. n = 6 independent samples for each group, error bars represent mean ± SD.

GSEA revealed that proteins for immune response and anti‐inflammation were downregulated (Figure 4A–D). This may contribute to reducing AGEs induced by the high‐glycemic environment, thus improving the microenvironment of the wound. To validate these findings, we quantitatively analyzed the levels of AGEs, RAGE, and inflammatory mediators. The AGEs content in the control group and T2DM mice kept increasing within 14 days. In contrast, polyphenol treatment reduced AGEs accumulation by Day 14 (*p* < 0.0001 vs. T2DM), even below healthy control levels, confirming that polyphenol can reverse the time‐course deposition of AGEs (Figure 4E). RAGE, as a transmembrane protein, delayed degradation resulted in a nonsignificant change at day 7 but a significant decrease at day 14 (Figure 4F). Polyphenol treatment reduced levels of multiple cytokines (IL‐4, IL‐6, IL‐10, TNF‐α), mitigating inflammatory storm‐induced tissue damage (Figure 4G–L), thereby priming the wound bed for regeneration. However, whether synergistic effects exist among these three polyphenols (EGCG, ECG, and EC) has not yet been thoroughly verified. In subsequent studies, we will conduct targeted experiments focusing on this direction to further elucidate the mechanism underlying their synergistic effect.

### 2.5 In Vivo Evaluation of the Wound Healing Effect of Polyphenol+ PDGF‐BB in Diabetic Mice

**FIGURE 5:**
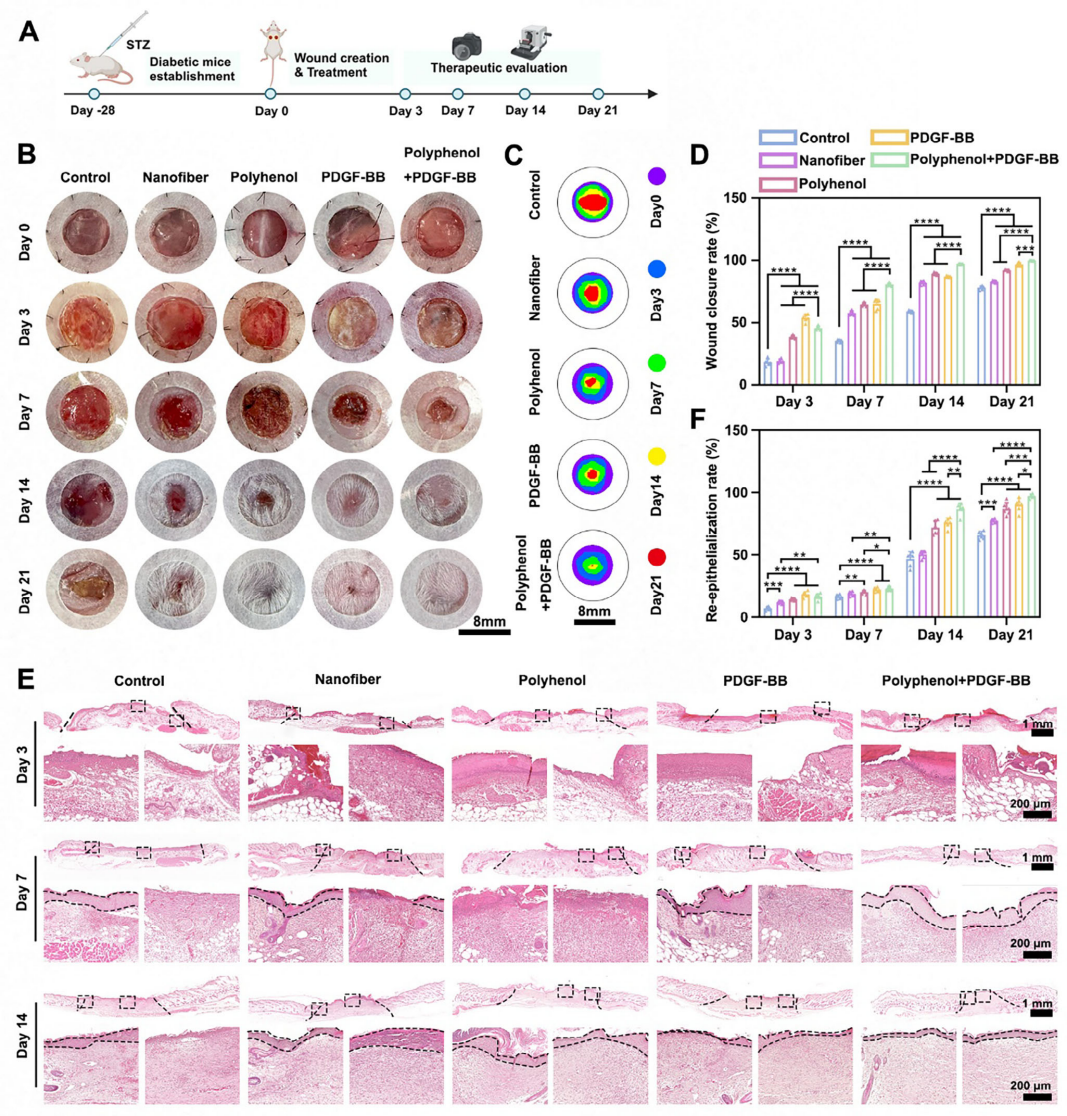
In vivo wound healing assessment. (A) Diagram showing the timeline of diabetic wound modeling, treatment, and treatment evaluation. (B) Representative images of skin wounds of Control, Nanofiber, Polyhenol, PDGF‐BB, and Polyhenol+PDGF‐BB groups at each indicated time point. (C,D) Quantification of wound closure rate in each group at 3, 7, 14, and 21 days. (E) Representative images of H&E staining of wounds in the Control, Nanofiber, Polyhenol, PDGF‐BB, and Polyhenol+PDGF‐BB groups on days 3, 7, and 14. (F) Re‐epithelialization rates of wounds after 3, 7, and 14 days of different treatments. ^*^ *p* < 0.05, ^**^ *p* < 0.01, ^***^ *p* < 0.001, ^****^ *p* < 0.0001. n = 6 independent samples for each group, error bars represent mean ± SD.

A chronic wound model was developed in diabetic mice to mimic the pathological conditions required to assess the double‐layer dressing efficacy (Figure 5A). In diabetic ICR mice with 8‐mm full‐thickness wounds, five interventions were tested: untreated control, Nanofibers, Polyhenol, PDGF‐BB, Polyphenol+PDGF‐BB (Figure 5B). While PDGF‐BB group showed early advantage at day 3, Polyhenol and Polyphenol+PDGF‐BB group achieved superior later‐phase healing due to initial wound bed cleansing (Figure 5C,D). At day 14, Polyphenol+PDGF‐BB group exhibited higher closure rates than PDGF‐BB (*p* < 0.0001), suggesting that there was a synergistic effect between Polyhenol and PDGF‐BB in wound repair. H&E staining was used to observe wound healing, and by day 3, each experimental group was accompanied by a large number of inflammatory cell infiltration. At day 14, the re‐epithelialization rate of nearly 100% was observed in the Polyphenol+PDGF‐BB double‐layer dressing group, followed by the PDGF‐BB group (Figure 5E,F).

### 2.6 Evaluation of Polyphenol+PDGF‐BB‐Mediated High Quality Wound Healing

**FIGURE 6:**
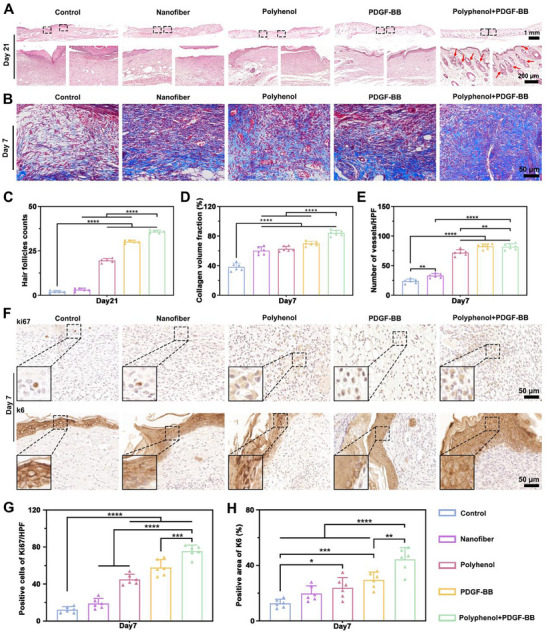
Evaluation of Polyhenol+PDGF‐BB‐mediated high‐quality healing. (A) Representative images of H&E staining at day 21. Red arrows: newborn skin appendages. (B) Masson's trichrome staining shows collagen deposition in the wounds of Control, Nanofiber, Polyhenol, PDGF‐BB, and Polyhenol+PDGF‐BB groups after 7 days of treatment. (C) Quantification of hair follicles. (D) Quantification of collagen volume fraction. (E) Quantification of vessels. (F) Immunohistochemical staining of wound Ki67 and K6 in Control, Nanofiber, Polyhenol, PDGF‐BB, and Polyhenol+PDGF‐BB groups after 7 days of treatment. (G,H) Quantification of Ki67 and K6. ^**^ *p* < 0.01, ^***^ *p* < 0.001, ^****^ *p* < 0.0001. n = 6 independent samples for each group, error bars represent mean ± SD.

Polyphenol+PDGF‐BB extensively regenerated skin appendages on day 21, which was better than the other groups, quantitatively counting 35.8 ± 0.8 regenerated in the wound area of Polyphenol+PDGF‐BB as compared to 2.0 ± 0.7 in the control group (Figure 6A,C). Masson's trichrome staining revealed abundant neovessels and dense collagen deposition in PDGF‐BB and Polyphenol+PDGF‐BB groups at day 7 (Figure 6B). Collagen volume fraction reached 84.1 ± 3.7% in bilayer vs. 38.4 ± 5.0% in controls (Figure 6D). Polyphenol+PDGF‐BB neovascularization quantification was 82.0 ± 4.9/ HPF not statistically different from that of the PDGF‐BB group (82.5 ± 4.1/ HPF), but higher than that of the control group (24.0 ± 3.6/ HPF) (Figure 6E). Given that wound healing depends critically on cellular proliferation and epithelial migration, we performed immunohistochemical staining to assess these processes. Ki67⁺ cells confirmed active proliferation in PDGF‐BB and Polyphenol+PDGF‐BB groups, with the Polyphenol+PDGF‐BB group exhibiting the highest proliferation activity (Figure 6F,G). Furthermore, the highest K6 expression was observed in the Polyphenol+PDGF‐BB group, indicating enhanced epithelial cell migration capacity and improved tolerance to mechanical stress (Figure 6F,H).

### 2.7 Mechanism of Inflammatory Microenvironment Regulation by Polyphenol+PDGF‐BB In Vivo

**FIGURE 7:**
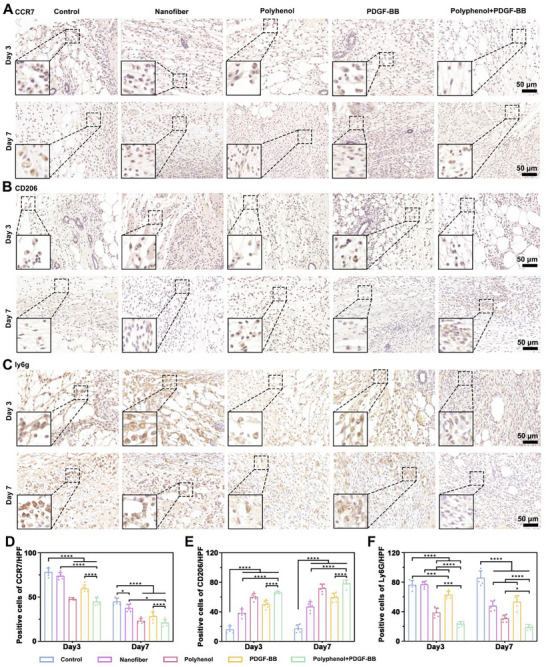
Inflammatory cellular regulation of Polyhenol+PDGF‐BB. Representative images of immunohistochemical staining of CCR7 (M1 macrophage), (B) CD206 (M2 macrophage), (C) Ly6G (a pan marker of monocytes, granulocytes, and neutrophils) in Control, Nanofiber, Polyhenol, PDGF‐BB, and Polyhenol+PDGF‐BB groups at 3 and 7 days. (D–F) Quantification of CCR7, CD206 and Ly6G. ^*^ *p* < 0.05, ^**^ *p* < 0.01, ^***^ *p* < 0.001, ^****^ *p* < 0.0001. n = 6 independent samples for each group, error bars represent mean ± SD.

Excessive inflammation during wound healing sustained release of proinflammatory mediators, resulting in endothelial damage, exacerbated edema, and local ischemia, which degrade collagen and growth factors and ultimately delay wound closure [35]. To assess the local inflammatory response, we examined the expression of key inflammatory markers in wound tissues. At 3 and 7 days, expression of CCR7 (M1‐type macrophage marker) was reduced in the Polyphenol and Polyphenol+PDGF‐BB groups, indicating a potent anti‐inflammatory effect of polyphenol (Figure 7A,D). At day 7, CD206⁺ M2 macrophages were increased in all groups except the control group compared with those at day 3 (Figure 7B,E). We also assessed Ly6g, a specific neutrophil marker associated with early immune activation and tissue damage when excessively retained in wounds. The number of Ly6g‐positive cells was markedly decreased in the Polyphenol+PDGF‐BB group compared to the control and nanofiber‐only groups, suggesting reduced neutrophil‐mediated injury and inflammation (Figure 7C,F).

**FIGURE 8:**
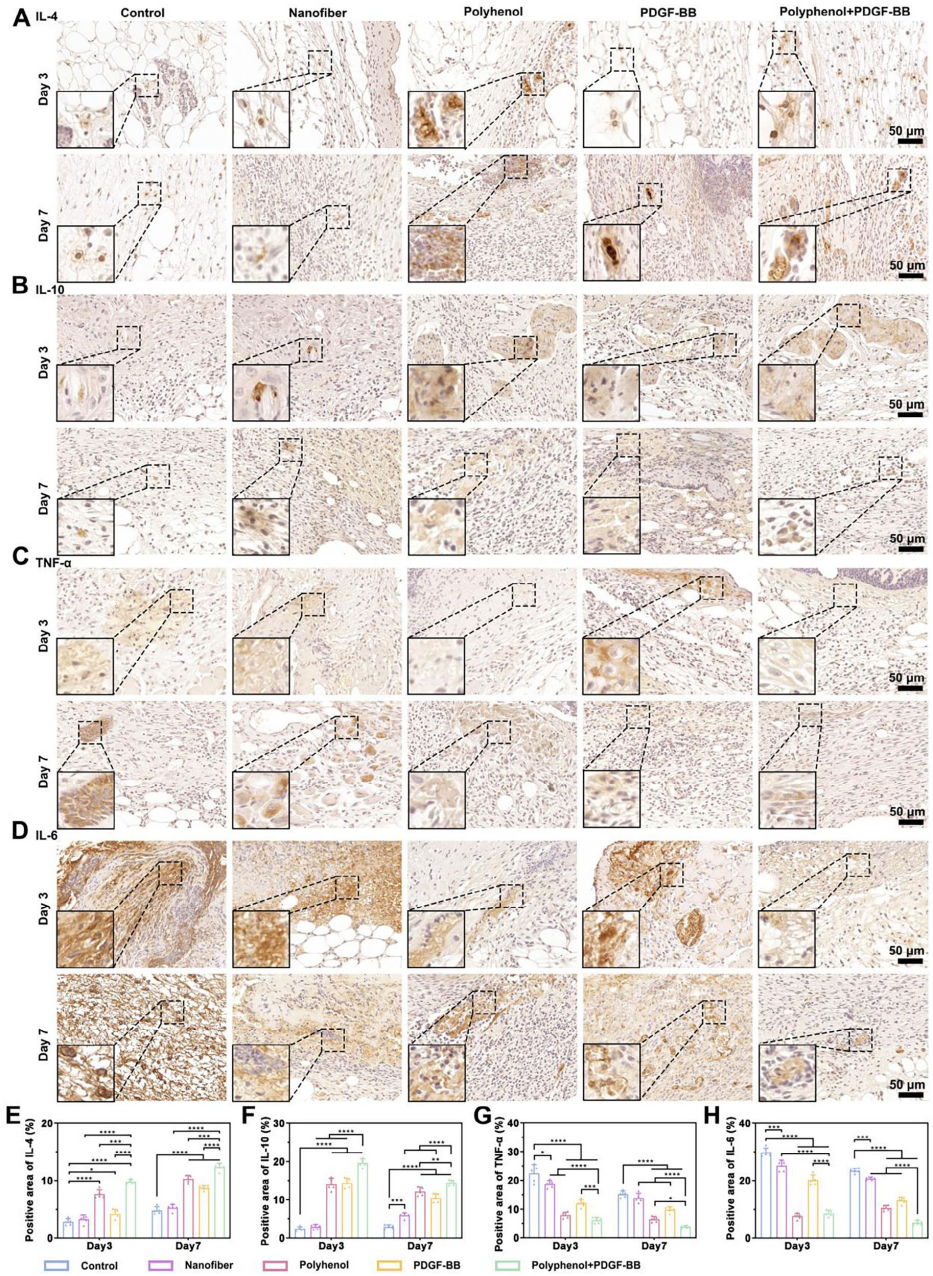
Expression of inflammatory and anti‐inflammatory factors during wound healing. (A) Representative images of immunohistochemical staining of IL‐4, (B) IL‐10, (C) TNF‐α, (D) IL‐6 in Control, Nanofiber, Polyhenol, PDGF‐BB, and Polyhenol+PDGF‐BB groups at 3 and 7 days. (E–H) Quantification of IL‐4, IL‐10, TNF‐α, and IL‐6. ^*^ *p* < 0.05, ^**^ *p* < 0.01, ^***^ *p* < 0.001, ^****^ *p* < 0.0001.

In our previous experiments, we performed subcutaneous injections of polyhenol into diabetic mice, and have verified that polyhenol has removed inflammatory factors from the base of the wound and acts like a protein scavenger (Figure 3). The anti‐inflammatory effect of polyhenol was further verified after polyhenol was used as a raw material to make a wound repair material. Immunohistochemical staining revealed that IL‐4 and IL‐10 (anti‐inflammatory cytokines) were elevated in the Polyphenol and Polyphenol+PDGF‐BB groups on day 3, and continued to increase by day 7 (Figure 8A,B,E,F). In contrast, the control group exhibited persistently low levels of these cytokines. Meanwhile, the expression of pro‐inflammatory cytokines TNF‐α and IL‐6 remained elevated in the control group at day 3 and day 7. Notably, their expression was lower in the Polyphenol+PDGF‐BB group compared to the Polyphenol group on day 7 (Figure 8C,D,G,H), suggesting a synergistic anti‐inflammatory effect of Polyphenol and PDGF‐BB, which collectively suppress excessive inflammation and promote tissue regeneration and remodeling.

## 3 Conclusion

In this study, we successfully developed a temporally regulated bilayer wound dressing based on the concepts of 1. providing a healthy wound bed and 2. enhancing regenerative activity. The bottom layer, a GelMA/Polyphenol cryohydrogel, acts as a “cleaner” by scavenging AGEs and remodeling the wound microenvironment. The top layer, composed of PCL/PDGF‐BB nanofibers, functions as a “regenerative engine”, continuously releasing growth factors to promote vascular regeneration, granulation tissue formation, and collagen deposition. Our findings demonstrated that the polyphenol+PDGF‐BB group outperformed other groups in improving the inflammatory wound environment, accelerating healing, and enhancing the quality of regenerated tissue. This wound composite system is expected to provide a highly effective, safe, and translatable novel therapeutic option for chronic diabetic wounds, with significant clinical impact potential.

## 4 Experimental Section

### 4.1 Materials and Reagents

PCL (catalog no. 440744), pluronic F‐127 (catalog no. 9003‐11‐6), ammonium persulfate (catalog no. 248614), TEMED (catalog no. T9281) were purchased from Sigma–Aldrich (St. Louis, USA). Recombinant Human PDGF‐BB (catalog no. C199‐50) was ordered from NovoProtein (Zhejiang, China). EGCG (catalog no. SR3289), ECG (catalog no. ECG), EC (catalog no. E22433) were ordered from HARVEYBIO (Beijing, China). GelMA was obtained from Wenzhou Shuhe Biological Technology Co., ltd (Zhejiang, China). Dulbecco's modified eagle medium (catalog no. 11965118), fetal bovine serum (catalog no.10099‐141C), and Penicillin‐streptomycin (catalog no. 15140122) were purchased from Gibco (Shanghai, China). Mouse Receptor for Advanced Glycosylation End Products ELISA Kit (catalog no. JL20579‐96T) and Mouse Advanced Glycosylation End Products ELISA Kit (catalog no. JL10691‐96T) were purchased from jianglai (Shanghai, China). Mouse TNF alpha PicoKine ELISA Kit (catalog no. EK0527), Mouse IL‐10 PicoKine ELISA Kit (catalog no. EK0417), Mouse IL‐6 PicoKine ELISA Kit (catalog no. EK0411), Mouse IL‐4 PicoKine ELISA Kit (catalog no. EK0405) were purchased from BOSTER (Wuhan, China).

### 4.2 Preparation of GelMA/Polyphenol Frozen Hydrogels

Three polyphenols (EC, ECG, and EGCG) were dissolved in PBS in three equal parts at a total concentration of 1 mg/mL. This solution was mixed with 0.75% (w/v) GelMA, 0.1 g/mL APS, and 1.29 µL/mL TEMED under ice‐cooled conditions to minimize bubble formation. The mixed solution was quickly transferred to a silica gel mold with a diameter of 8 mm and a depth of 3 mm, and placed in a −20°C for 12 h of freezing and crosslinking overnight.

### 4.3 Preparation of PCL/PDGF‐BB Nanofiber Membranes

PCL (1 g) and GelMA (0.5 g) were dissolved in 10 mL trifluoroethanol with 0.5% (v/v) glacial acetic acid to optimize solution conductivity. Pluronic F127 (1% w/v) was added to minimize bead defects during electrospinning. Sufficiently dissolved to obtain the spinning stock solution. 50 µg of PDGF‐BB was reconstituted in 10 mL spinning solution. Electrospinning was performed at 0.5 mL/h flow rate, 18 kV voltage, 20 cm collector distance, and 3000 rpm rotation. Fibers collected on aluminum foil were punched into 8‐mm discs. Avoid vigorous stirring during the preparation of the PDGF‐BB electrospinning solution. Optimize electrospinning parameters to prevent denaturation caused by localized heat generation during spinning. Immediately after preparation, vacuum seal in sterile aluminum foil pouches to prevent oxidative inactivation of PDGF‐BB. Sterilize using ethylene oxide, label each sealed pouch with its batch number, and ensure each pouch contains only one scaffold to prevent cross‐contamination. Store sterilized and packaged scaffolds in a 4°C light protected refrigerator (humidity controlled at 30%–40%).

### 4.4 Characterization of Materials

The 0.75% GelMA, GelMA/Polyphenol, PCL/PDGF‐BB were sputter‐coated with platinum for 90s (Leica, EM ACE600). SEM (Hitachi, SU8010) was used to observe the structure of the material, and then further quantified the average pore size of the fibers by image J. A universal testing machine (Model: Instron‐5944) was used to perform tensile tests on film samples. The samples were cut into stripes (25 mm × 10 mm) before testing. The tensile rate was set to 10 mm/min, and the stress–strain curve was recorded to calculate key mechanical parameters. PCL and PCL/PDGF‐BB were cut into 8 mm circles and fixed on glass slides. A static contact angle goniometer (Theta, Sweden) was preheated and calibrated prior to measurement. A 5 µL aliquot of ultrapure water was dispensed onto 5 distinct positions on the membrane surface. After the water droplets stabilized, images were captured continuously from 0 to 60 s. The average contact angle was calculated using the corresponding software.

### 4.5 In Vitro Biocompatibility Assessment

L929 fibroblasts were cultured in high‐glucose DMEM supplemented with 10% FBS and 1% penicillin‐streptomycin (37°C, 5% CO_2_). L929 cells were inoculated into 96‐well plates with 5 × 10^3^ cells per well, after 24 h of culture, the cells were co‐cultured with GelMA/polyphenol, PCL/PDGF‐BB, and polyphenol+PDGF‐BB for 1 and 3 days, respectively, and the cells were stained with live/dead reagent (L3224, ThermoFisher, USA). Cell viability was observed using an inverted fluorescence microscope (Axio Vert.A1, ZEISS, Germany). Similarly, 5 × 10^3^ cells were inoculated into 96‐well plates and incubated with the material for 1, 3, and 5 days, respectively. CCK‐8 (C0037, Beyotime, China) was employed to assess the cell proliferation capacity, using a microplate spectrometer for analysis.

### 4.6 In Vitro of Drug Release

Immerse the double‐layered material in PBS buffer at pH 6.0, with a solution height of only 1 mm. Incubate the sample in a constant‐temperature shaking incubator at 37°C (50 rpm). Collect the buffer solution at predetermined intervals and replenish with fresh buffer solution. The Folin‐Ciocalteu method was used to determine the polyphenol release from the GelMA layer at time points including 0, 6, 12, 24, 36, 48, 60, and 72 h. An ELISA kit was used to determine the PDGF‐BB release from the PCL nanofiber membrane at time points including 1, 3, 5, 7, 10, and 14 d.

### 4.7 Establishment of Type II Diabetic Mouse Model

Approved by the Animal Ethics Committee of the Wenzhou Institute of Biological Sciences (Wenzhou Institute of Biomaterials and Technology) (approval number: WIUCAS24040402). Female ICR mice (8–10 weeks, 30 g) were fed a high‐fat diet for 4 weeks and administered intraperitoneal injections of streptozotocin (STZ, 40 mg/kg/day) for 7 consecutive days. Seven days after the injection, fasting blood glucose and body weight were monitored daily via tail vein sampling. Mice exhibiting body weight ≥ 35 g and fasting blood glucose ≥ 16.5 mmol/L for 3 days were considered diabetic. Individuals with >15% glucose fluctuation were excluded.

### 4.8 Polyphenol Subcutaneous Injection Test

ICR mice were randomized into three groups: healthy controls (no intervention), T2DM group (daily subcutaneous injection of 100 µL PBS, and Polyphenol group (100 µL polyphenol solution was injected subcutaneously daily at the same site) for 14 days. Dorsal subcutaneous tissues were harvested post‐euthanasia, rinsed with ice‐cold PBS, homogenized at 1:9 (w/v). Supernatants collected after centrifugation (5000×g, 10 min) were subjected to ELISA quantification of AGEs, their receptor (RAGE), and cytokines (IL‐6, TNF‐α, IL‐4, IL‐10). Another tissue samples were fixed in 4% paraformaldehyde for H&E and Masson's trichrome staining, and proteomic sequencing was performed on day 7.

### 4.9 ELISA Assay

Target molecules were quantified using commercial ELISA kits per manufacturer's protocols. Briefly, six‐point serial dilutions of standards were prepared in universal assay diluent. After 10 min equilibration at room temperature, 100 µL of standards, samples, or blank controls were loaded into pre‐coated wells. Following 60 min incubation at 37°C under sealing film, liquids were discarded, and biotinylated detection antibody was directly added without washing. After 30 min incubation at 37°C, plates underwent three washing cycles. Horseradish peroxidase‐conjugated streptavidin (100 µL/well) was then added for 30 min incubation at 37°C. Post five washes, 90 µL TMB substrate solution triggered chromogenic reaction (15 min, 37°C in dark). The reaction was terminated, and at 450 nm measured immediately for concentration extrapolation via standard curves.

### 4.10 In Vivo Diabetic Wound Healing Test

The wound healing efficacy of the bilayer construct was evaluated in diabetic ICR mice randomized into five groups (n = 6/group): untreated controls (Control), PCL nanofiber monotherapy (Nanofiber), polyphenol‐loaded 0.75% GelMA cryogel (GelMA/Polyphenol), PDGF‐BB‐loaded nanofibers (PDGF‐BB), and bilayer combination (Polyphenol+PDGF‐BB). Under isoflurane anesthesia, bilateral 8‐mm full‐thickness excisional wounds were created on the dorsal skin. Silicone splints anchored with 6‐0 sutures prevented wound contraction. Wound closure was recorded by camera, and the percentage of healing was calculated by ImageJ as [1‐(Area_t_/Area_0_)] × 100.

### 4.11 Histological Observation

Mice were sacrificed at 4 time points on days 3, 7, 14, and 21, and tissues were taken for histological morphology analysis to observe the macroscopic healing of tissues, collagen deposition, re‐epithelialization, stratum corneum formation, and inflammatory cell infiltration, and to assess the quality of traumatic healing at the histological level. The collected tissues were fixed. The wax blocks were cut into 5‐µm‐thick sections, and HE staining with Masson trichrome staining was carried out for each group of sections. By analyzing the HE staining results, the reepithelialization rate was calculated as (length of reepithelialization/total width of initial wound) ×100%. Using image J for quantification.

### 4.12 Immunohistochemical Staining

Sections of traumatized tissue were deparaffinized, rehydrated, and subjected to sodium citrate antigen repair. Subsequently, the sections were closed with a BSA solution of 5% goat serum for 1 h. Next, the sections were placed at 4°C and incubated overnight with primary antibodies containing Ki67 (1:500, ab16667, Abcam), K6 (1:500, bsm‐60235R, Bioss), CCR7 (1:500, ab253187, Abcam), CD206 (1:500, PA5‐101657, Invitrogen), Ly6G (1:300, ab238132, Abcam), IL‐4 (1:250, TA5142M, Abmart), IL‐10 (1:250, TD6894, Abmart), IL‐6 (1:250, TD6087, Abmart), and TNF‐α (1:250, TA7014, Abmart). On the second day, the sections were treated with HRP‐labeled secondary antibody for 1 h at room temperature, followed by staining reaction by DAB (1:20). Finally, scanning and analysis of the sections were completed using a pathology section scanner, and the amount of positive expression in the tissue sections was quantified using image J.

### 4.13 Statistical Analysis

All statistical results were presented as means ± SD. Based on statistical power analysis, the animal sample size for this study was n = 6 per group. The figure legends indicate the number of replicates (n); each data point corresponds to an independent biological replicate or experiment. Data were analyzed using GraphPad Prism 8.0 software, and differences between groups were analyzed by one‐way analysis of variance followed by Tukey's multiple comparison test. ^*^ *p* < 0.05, ^**^ *p* < 0.01, ^***^ *p* < 0.001, and ^****^ *p* < 0.0001 were considered statistically significant.

## Conflicts of Interest

The authors declare no conflicts of interest.

## Supporting information




**Supporting File**: advs73634‐sup‐0001‐SuppMat.docx.

## Data Availability

The data that support the findings of this study are available from the corresponding author upon reasonable request.
