# Supplementary material for: A Time‐Programmed Bilayer Wound Dressing for Dynamic Microenvironment Modulation and Full‐Thickness Regeneration in Diabetic Wounds
Source: Adv Sci (Weinh). 2026 Jan 8;13(10):e12425. doi: 10.1002/advs.202512425 (PMC12915078; doi:10.1002/advs.202512425)
Supplement: Supplementary file 1 — Supporting File: advs73634‐sup‐0001‐SuppMat.docx. [file ADVS-13-e12425-s001.docx]

**Supplementary Data**

**A Time-Programmed Bilayer Wound Dressing for Dynamic Microenvironment Modulation and Full-Thickness Regeneration in Diabetic Wounds**

Lei Yi ^1,2 §^, Wanqian Li ^2,3, §^, Ying Duanmu ^4, §^, Zihan Zhang ^2^,

Shixuan Chen, Shichu Xiao ^4, *^, Lei Du ^2, *^, Miaomiao Wei ^2, *^

^1^ Department of Burn, Ruijin Hospital, Shanghai Jiao Tong University School of Medicine, Shanghai, China

^2^ Zhejiang Engineering Research Center for Tissue Repair Materials, Wenzhou Institute, University of Chinese Academy of Sciences, Wenzhou, Zhejiang, 325000, China

^3^ Department of Burn and Plastic Surgery, Department of Wound Repair Surgery, Affiliated Hospital of Nantong University, Nantong, Jiangsu, 226001, China

^4^ Department of Burn Surgery, The First Affiliated Hospital of Naval Medical University, Shanghai,200433，China

^§^ These authors contributed equally to this work.

* Corresponding Email:

huangzhuoxiao4@hotmail.com (S. Xiao)

[dulei@ucas.ac.cn](mailto:dulei@ucas.ac.cn) (L. Du);

miaomiaoweiz@163.com (M. Wei);

**
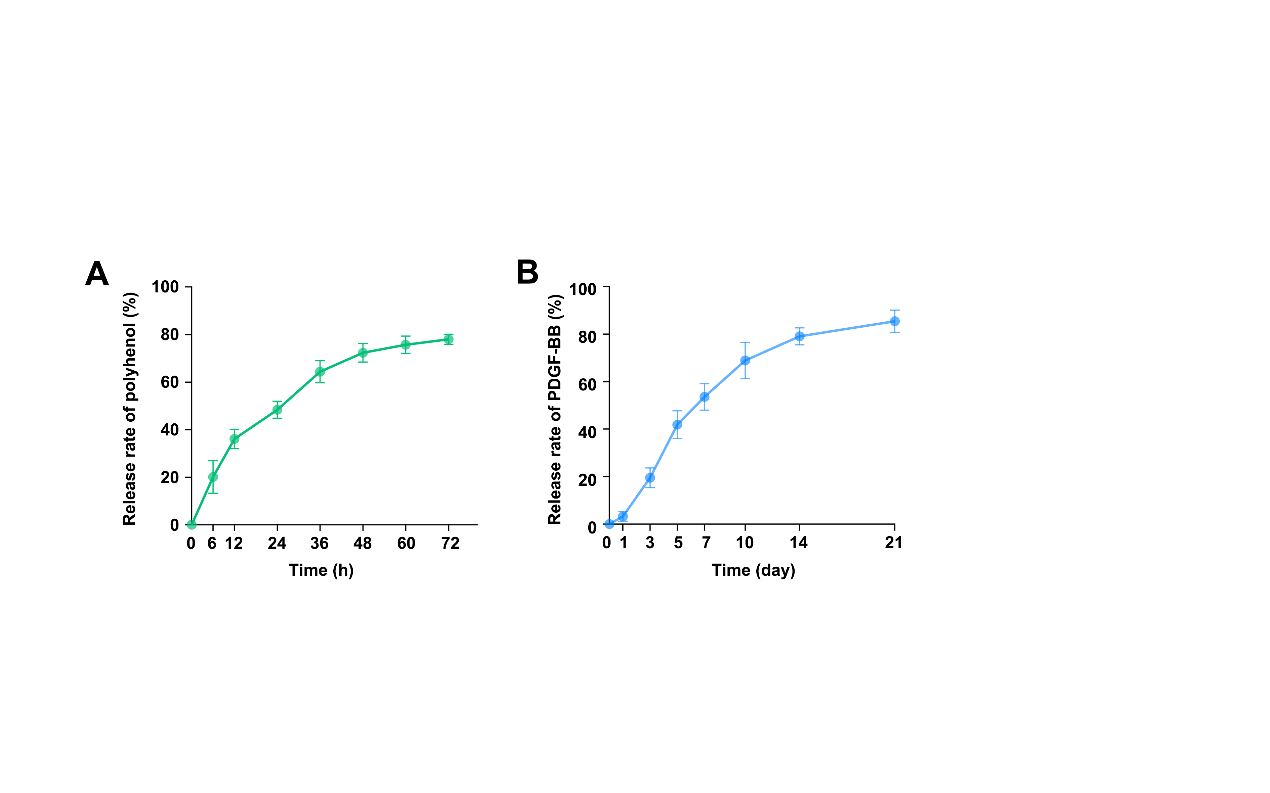
**

**Supplementary Figure 1.** A, B) *In vitro* release kinetics of polyphenols and PDGF-BB. n=3, error bars represent mean ± SD.


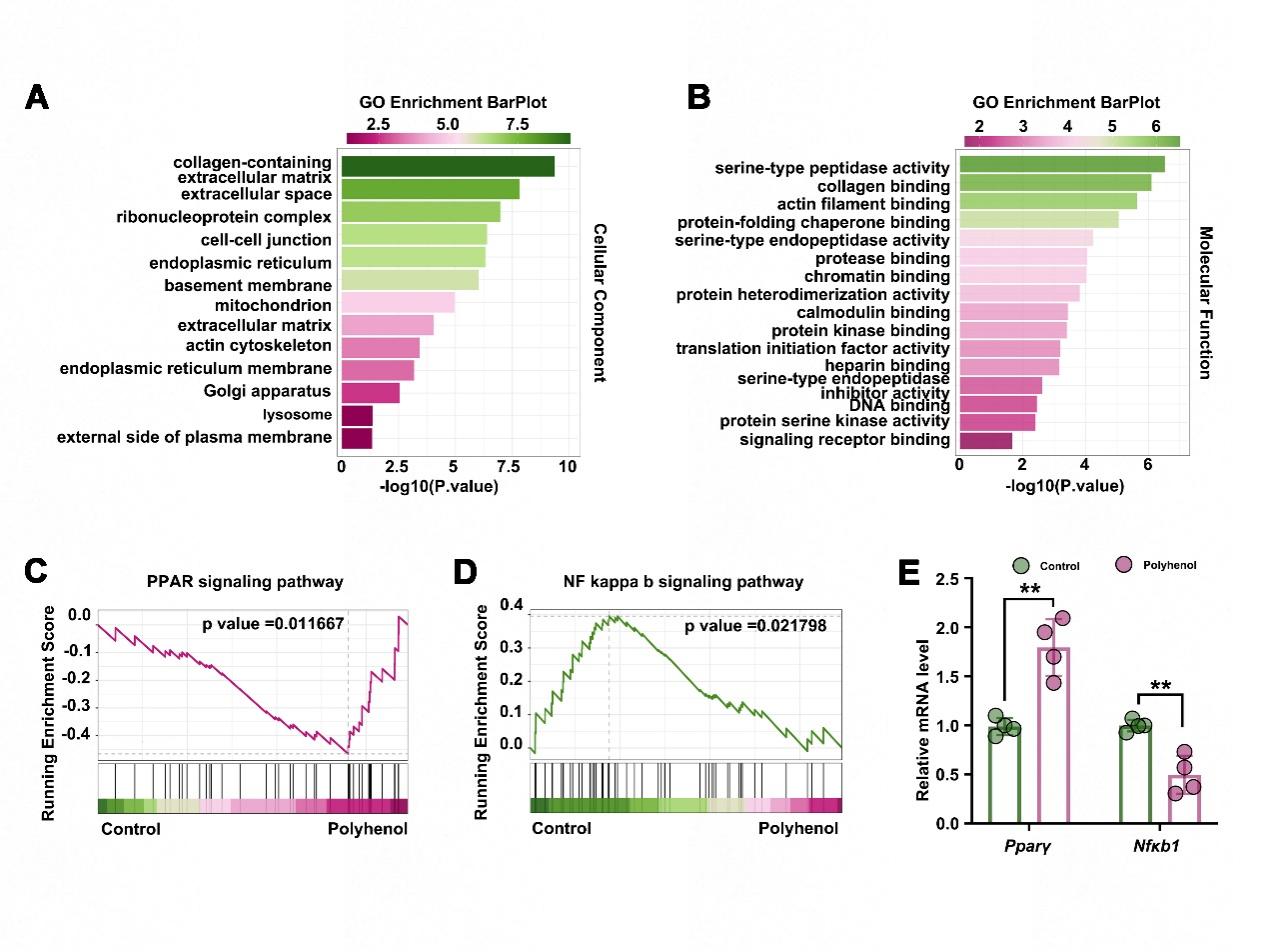


**Supplementary Figure 2.** A, B) GO functional enrichment analysis including Cellular Component, Molecular Function. GSEA of C) PPAR signaling pathway, D) NF Kappa b signaling pathway, E) qPCR validation of relative mRNA expression levels for key genes *Pparγ* and *Nfkb1*. ***p* < 0.01, n=4, error bars represent mean ± SD.
